# Integration of Systemic Therapy and Stereotactic Radiosurgery for Brain Metastases

**DOI:** 10.3390/cancers13153682

**Published:** 2021-07-22

**Authors:** Raees Tonse, Martin C. Tom, Minesh P. Mehta, Manmeet S. Ahluwalia, Rupesh Kotecha

**Affiliations:** 1 Department of Radiation Oncology, Miami Cancer Institute, Baptist Health South Florida, Miami, FL 33176, USA; Mohammed.tonse@baptisthealth.net (R.T.); MartinTo@baptisthealth.net (M.C.T.); MineshM@baptisthealth.net (M.P.M.); 2 Herbert Wertheim College of Medicine, Florida International University, Miami, FL 33199, USA; Manmeeta@baptisthealth.net; 3 Department of Medical Oncology, Miami Cancer Institute, Baptist Health South Florida, Miami, FL 33176, USA

**Keywords:** stereotactic radiosurgery, chemotherapy, targeted therapy, immunotherapy, brain metastases

## Abstract

**Simple Summary:**

In the multi-modal treatment of brain metastasis (BM), the role of systemic therapy has undergone a recent revolution. Due to the development of multiple agents with modest central nervous system penetration of the blood-brain barrier, targeted therapies and immune checkpoint inhibitors are increasingly being utilized alone or in combination with radiation therapy. However, the adoption of sequential or concurrent strategies varies considerably, and treatment strategies employed in clinical practice have rapidly outpaced evidence development. Therefore, this review critically analyzes the data regarding combinatorial approaches for a variety of systemic therapeutics with stereotactic radiosurgery and provides an overview of ongoing clinical trials.

**Abstract:**

Brain metastasis (BM) represents a common complication of cancer, and in the modern era requires multi-modal management approaches and multi-disciplinary care. Traditionally, due to the limited efficacy of cytotoxic chemotherapy, treatment strategies are focused on local treatments alone, such as whole-brain radiotherapy (WBRT), stereotactic radiosurgery (SRS), and resection. However, the increased availability of molecular-based therapies with central nervous system (CNS) penetration now permits the individualized selection of tailored systemic therapies to be used alongside local treatments. Moreover, the introduction of immune checkpoint inhibitors (ICIs), with demonstrated CNS activity has further revolutionized the management of BM patients. The rapid introduction of these cancer therapeutics into clinical practice, however, has led to a significant dearth in the published literature about the optimal timing, sequencing, and combination of these systemic therapies along with SRS. This manuscript reviews the impact of tumor biology and molecular profiles on the management paradigm for BM patients and critically analyzes the current landscape of SRS, with a specific focus on integration with systemic therapy. We also discuss emerging treatment strategies combining SRS and ICIs, the impact of timing and the sequencing of these therapies around SRS, the effect of corticosteroids, and review post-treatment imaging findings, including pseudo-progression and radiation necrosis.

## 1. Introduction

Brain metastases (BM) represent the most common intracranial neoplasm in adults and occur in approximately 20–40% of all cancer patients [1]. The most common primary tumors in patients with BM are lung, breast, melanoma, colorectal, and renal, and these tumors are associated with a median survival time of 6–12 months [1]. BMs are distributed along regions of the brain with rich blood flow, with 80% occurring in the cerebral hemispheres, primarily at the grey-white junctional border [2]. Patients often develop symptoms consequential to the location of the tumor, either by direct tumor infiltration of critical functional regions, or due to the associated mass effect. Radiation therapy (RT), in the form of stereotactic radiosurgery (SRS) or whole-brain radiotherapy (WBRT) is considered a mainstay anticancer modality in the treatment of BM from solid tumors [3]. However, the management of BM is based on patient and tumor-specific variables, such as tumor histology, performance status, prognosis, extent of extracranial disease, presence of targetable actionable mutations, number of lesions, volume of disease, symptoms, and patient preference [3].

The role of systemic therapy in the treatment of BM is evolving. Previously, its role was restricted due to variable CNS penetration of the blood-brain barrier (BBB) and limited activity [4]. Targeted therapies with greater CNS penetration and improved efficacy have emerged in parallel with the identification of driver mutations, which have led to advances in drug discovery and development [5]. Immune checkpoint inhibitors (ICIs) represent another significant advancement in systemic therapy options for BM, as they have shown promising CNS activity in subsets of patients [5]. As a result, BM can now be managed with systemic therapy either prior to, concomitantly, or after RT, and various combinations of RT with systemic therapies are being explored to improve both local and extracranial disease control, as well as overall survival (OS). This necessitates effective management strategies from multidisciplinary teams, as treatment decisions must balance the risk of recurrence/progression with treatment-related side effects. Previous reviews have compiled data from retrospective and prospective studies of combination approaches [5,6]. However, in this review, we summarize the data from recent studies and clinical trials supporting the use of BM-directed systemic therapies, such as chemotherapy, targeted therapy, and immunotherapy, that have been completed or are currently being investigated, and their integration with SRS for the treatment of BM.

## 2. Modern Role for Stereotactic Radiosurgery

SRS is a specialized RT technique that delivers a single, high dose of radiation to the tumor. Although this treatment was previously used in tumors less than 3 cm in maximum dimension, fractionated approaches of up to five fractions are employed for moderate-large sized lesions or those in close proximity to critical structures, such as the brainstem, optic nerves, and optic chiasm.

SRS has undergone rapid technological development over the last decade and continues to evolve. Its success relies on submillimetric precision in target localization, which was previously achieved by invasive, fixed stereotactic head frames. Online cone-beam computed tomography (CT) scanning is now routinely used for precision localization in modern linac-based radiosurgical systems, reducing the need for skeletal fixation and permitting fractionation. The CyberKnife^®^ (Accuray Inc., Sunnyvale, CA, USA) and the Novalis ExacTrac X-Ray 6D (Brainlab, Munich, Germany) systems are two examples among a number of frameless image-guided stereotactic systems that do not require invasive cranial fixation. For photon-based SRS, a variety of systems are available; the most commonly used are the GammaKnife^®^ (Elekta AB, Stockholm, Sweden), CyberKnife^®^ (Accuray Inc., Sunnyvale, CA, USA) and linac-based systems, all of which have similar efficacy. Modern GammaKnife^®^ systems also permit the use of mask-based fixation approaches [7]. Particle radiosurgery has the potential for radiobiological benefits from the higher radiobiological effectiveness, as well as dosimetric advantages including decreased normal tissue exposure and improved dose homogeneity as compared to photon based SRS [8]; however, the dosimetric benefits are generally restricted to large, complex shaped targets, as smaller targets are in fact treated with superior dosimetric considerations using photon systems [9]. Presently there is a paucity of data on the use of particle therapy in the context of radiosurgery; however, future developments are expected to improve our understanding of these emerging technologies, particularly as the number of particle therapy facilities continue to grow.

SRS is commonly utilized for patients with a disease-specific graded prognostic assessment (DS-GPA) [10] score over 2, low intracranial disease burden, and minimal neurological symptoms. When compared to WBRT, a phase III study reported that SRS produces a similar OS with less decline in neurocognitive function (WBRT plus SRS 53% vs. 20% SRS alone), but with a significantly increased risk of intracranial relapse [11]. SRS is preferred for patients with a limited number of BM (4 or fewer lesions) based on the results from randomized trials [12,13]. The radiation doses are based on tumor dimension, <2 cm, 2.1–3 cm and >3 cm are 24 Gy, 18 Gy and 15 Gy, respectively, based on the Radiation Therapy Oncology Group (RTOG) 90-05 study [14]. The efficacy of SRS appears to be independent of the primary tumor type, as radioresistant tumors (i.e., renal cell carcinoma and melanoma) have similar control rates as radiosensitive tumors (i.e., breast cancer and lung cancer) [15,16]. Single fraction SRS is not recommended for lesions >4 cm due to an unacceptable level of toxicity [17]. However, hypofractionated SRS (HF-SRS) or staged SRS can be considered for larger lesions [17]. Fractionated SRS is typically delivered to 25–30 Gy over 3–5 fractions and is considered for lesions close to critical structures, such as the brainstem or the optic apparatus. Some centers utilize the concept of low overall intracranial disease burden based on total volume of all brain metastases (<15–30 cc) to select patients to be treated with SRS; however, this parameter has not been defined adequately and requires prospective validation [4].

In the context of post-operative RT, SRS has replaced WBRT in most instances, but the issue of the optimal interval between surgery and SRS remains ill-defined [18,19]. Further, several reports suggest that pre-operative SRS reduces the risk of meningeal metastases and symptomatic radiation necrosis (RN) compared to post-operative SRS [20,21]. Pre-operative SRS allows for better target volume delineation, as opposed to a poorly-defined irregularly shaped surgical cavity in the post-operative setting. It also allows for better tumor control by reducing the intra-operative seeding of viable tumor cells outside the treated cavity, hence decreasing the risk of leptomeningeal disease [22]. The rate of symptomatic RN may be reduced with pre-operative SRS as target delineation is better, less normal brain is irradiated, and the majority of the irradiated tissue is resected after SRS [21]. One major limitation of pre-operative SRS is the lack of pathological confirmation prior to SRS. Moreover, select reports demonstrate that pre-operative SRS has the potential to lead to increased wound healing complications [23].

In the post-operative setting, high dose HF-SRS provided greater local control (LC)—defined as radiographic evidence of stable disease, partial response, or complete response, as compared to lower biological effective dose (BED) regimens (95% vs. 59%) [24]. For example, 25 Gy in 5 fractions (BED10 of 37.5 Gy) was not adequate to control microscopic disease as compared to 30 Gy in 5 fractions (BED10 > 48 Gy) which had excellent tumor bed control. Similarly, another study reported that HF-SRS after resection of BM was well tolerated and had improved LC with BED10 ≥ 48 (i.e., 30 Gy/5 fractions and 27 Gy/3 fractions) [25].

The LC rates following SRS for 5 or more intracranial lesions are comparable to those for fewer lesions [26]; however, these patients continue to experience a high rate of distant intracranial failure, and therefore alternative treatment strategies, such as hippocampal-avoidant whole brain radiotherapy (HA-WBRT), should be considered. There is evolving evidence that primary SRS alone can be used in select patients with >10 lesions [27]. A phase III randomized trial of SRS vs. WBRT in 72 patients with 4–15 BMs (NCT01592968) has also been presented, and demonstrated that SRS was associated with a reduced risk of neurocognitive deterioration relative to WBRT without compromising OS, but clearly with higher risk of intracranial relapse [28]. A prospective phase III trial (NCT03550391) will compare stereotactic radiosurgery with HA-WBRT plus memantine for 5–15 brain metastases.

## 3. Stereotactic Radiosurgery and Systemic Therapies

### 3.1. Chemotherapy

**Figure 1:**
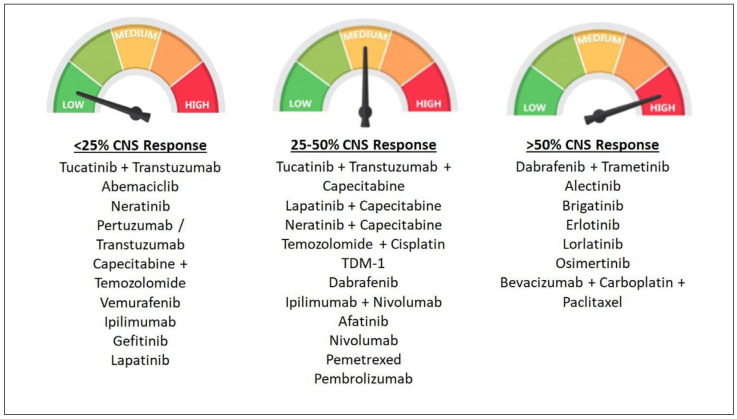
Categorization of various chemotherapeutic drugs or regimens based on CNS response (defined as proportion of patients with stable disease, partial response, or complete response) into low (<25%), medium (25–50%), and high (>50%).

Most traditional chemotherapeutic drugs have variable, but limited, BBB penetration (Figure 1), and are usually used in conjunction with local treatments like RT. Several phase II studies evaluating temozolomide (TMZ) and WBRT have shown that this combination increases LC, but not OS [29,30]. Platinum and pemetrexed have been used for the treatment of non-small cell lung cancer (NSCLC) BM, either alone or in combination with other treatments [31]. For patients with BM from breast cancer or melanoma, there is no particular chemotherapy regimen that has been shown to prolong survival.

There are limited data on the outcomes of concurrent chemotherapy with SRS for the treatment of BM. Cagney et al. reported the outcomes of patients treated with pemetrexed and SRS for lung cancer BM, and found that the combination was associated with a reduced likelihood of developing new brain metastases (*p* = 0.006) and a reduced need for brain-directed salvage RT (*p* = 0.005) [32]. However, the combination of pemetrexed and SRS was found to be associated with increase in radiographic RN (HR 2.70, 95% CI 1.09–6.70, *p* = 0.03). The authors concluded that patients who receive pemetrexed after brain-directed SRS tend to benefit from increased intracranial disease control at the potential cost of radiation-related RN. Shen and colleagues also demonstrated the safety of concurrent chemotherapy and SRS in 193 patients, of whom 37% were delivered with concurrent systemic therapy [33]. Kim and colleagues evaluated the outcomes in 1650 patients who presented with 2843 intracranial metastases [34]; among these, 445 patients (27%) were treated with SRS and concurrent systemic therapy. The risk of RN in those treated with SRS and concurrent systemic therapy was not increased as compared to SRS alone (6.6% and 5.3%); however, concurrent systemic therapy was linked to a higher rate of radiographic RN in lesions treated with upfront SRS and WBRT (8.7 vs. 3.7%, *p* = 0.04). Further study is warranted to explore whether symptomatic RN occurs more frequently in patients receiving pemetrexed along with SRS, and detailed analyses of other systemic therapy combinations are clearly needed to inform clinical practice.

### 3.2. Targeted Therapies

The use of targeted therapies in patients with actionable alterations represents a popular topic in BM research. Patients with these specific molecular subtypes respond to targeted therapies at higher rates than to chemotherapeutic agents or ICIs. As patients with BM have traditionally been excluded from clinical trials assessing systemic therapies in BM patients, the role of these systemic treatments, particularly when used in conjunction with SRS for BM, is unclear. This section summarizes the data regarding the combination of various targeted therapies with SRS.

#### 3.2.1. Anti-Human Epidermal Growth Factor Receptor 2 (HER-2) Drug Conjugates and Anti HER-2 Tyrosine Kinase Inhibitors (TKIs)

First-generation anti-HER2 agents have limited BBB permeability and minimum intracranial activity. Newer-generation agents have better CNS penetration, intracranial activity, and the potential for combinatorial synergy with SRS. In one retrospective series of HER2+ breast cancer patients treated with SRS with or without lapatinib [35], LC was significantly higher in the lapatinib group. In another series, the addition of concurrent lapatinib to SRS was associated with an improved rate of complete response compared to SRS alone, without an increased risk of RN [36]. Parsai et al. reported improved survival (27.3 vs 19.5 months, *p* = 0.03) when lapatinib was used concurrently with SRS, with reduction in local failure at 12 months (5.7% vs 15.1%, *p* < 0.01) [37]. Not all studies have demonstrated such a benefit; in fact, the prospective RTOG 1119 trial, a randomized study of WBRT or SRS with or without concurrent lapatinib, showed no increase in 12-week overall response rate with combined treatment [38]. Therefore, well-controlled clinical trials are clearly needed to generate high-quality evidence to support such combinatorial use.

The experience with small numbers of patients suggests that combining SRS with trastuzumab emtansine (T-DM1) might result in high rates of RN. In one study, SRS was given concurrently with T-DM1 in 4 patients, and sequentially in 8 patients [39]. The concurrent group had a 50% rate of RN while the sequential group had a 28.6% rate of RN. In a separate report, RN was observed in 40% of patients that received T-DM1 [40]. In contrast, Mills et al. reported that the combination of SRS and T-DM1 was well tolerated, with only 3% of patients reporting RN [41]. Hence, prospective studies to evaluate the ideal dose of SRS and timing of T-DM1 are warranted.

#### 3.2.2. Epidermal Growth Factor Receptor (EGFR)-TKIs

SRS can disrupt the BBB, thereby increasing the CNS penetration of EGFR-TKIs to penetrate the BBB [39]. Therefore, combinatorial SRS and TKIs approaches may prove to be useful, but few trials have tested this robustly. In part, the challenge lies in the fact that SRS alone yields very high LC rates, and therefore, any synergistic benefit from TKIs would likely not readily be observed [40]. However, SRS is an ineffective modality for controlling microscopic disease in the brain, which TKIs might be able to control, and therefore, intracranial disease control (via reduction in distant intracranial failures) could prove to be the most relevant endpoint for such combinatorial trials. An intriguing possibility is whether such improved intracranial control could translate to meaningful survival improvements, and this would only occur in clinical scenarios where patients have high risks of dying from intracranial progression. The role of EGFR in modulating radiosensitivity has been documented in numerous preclinical and in vitro studies [42]. One multicenter retrospective report underscored this possibility following the treatment of EGFR-mutated NSCLC BM with either SRS followed by EGFR-TKI, WBRT followed by EGFR-TKI, or EGFR-TKI followed by SRS or WBRT [43]. Combinatorial SRS and EGFR-TKI yielded the longest OS, whereas EGFR-TKI alone had the lowest survival (46 months vs. 25 months). In another study, patients with NSCLC BM were divided into two categories depending on the use of EGFR-TKI (at the time of the first SRS or post-SRS within 3 weeks) versus patients who received SRS alone. Patients receiving EGFR-TKI concurrently or soon after SRS had significantly longer OS (25.5 vs. 11.0 months), in spite of equivalent LC [44]. LC has been shown to be superior in some retrospective studies of patients with NSCLC BM treated with SRS in combination with TKIs such as gefitinib, erlotinib, or icotinib; in one study, the LC rate was 83.3% in the combination therapy group and 61.5% in the SRS monotherapy group [45]. In another report, intracranial control was improved in patients who received RT followed by icotinib versus icotinib alone (22.4 vs. 13.9 months, *p* = 0.043) [46].

Osimertinib, a third-generation EGFR TKI, has high CNS penetration [47] (Figure 1). In a retrospective study of NSCLC BM patients, Xie et al. compared the outcomes of patients treated with RT and osimertinib versus osimertinib alone [48]. There was no difference in time to treatment failure, progression-free survival (PFS), or OS between the groups. These limited data provide initial safety and possible efficacy signals. However, no level 1 evidence (randomized controlled clinical trial) exists to support the routine combinatorial approach of SRS plus EGFR TKIs in NSCLC BMs, and such studies are critically needed [49].

#### 3.2.3. Anaplastic Lymphoma Kinase (ALK) Inhibitors

Rearrangements of the ALK gene have been found in around 3–7% of NSCLC cases [50]. Concurrent RT and ALK-TKIs has also been shown in preclinical studies to have a synergistic effect on tumor growth and microvessel density, possibly resulting in better LC [50,51]. A multi-institutional study [52] showed that patients treated with SRS or WBRT and ALK inhibitors (crizotinib, ceritinib, alectinib) had a median OS of 49.5 months (95% CI, 29.0 months compared to not reached) and a median intracranial progression-free survival of 11.9 months (95% CI, 10.1 to 18.2 months). Prognostic factors such as Karnofsky performance status (KPS) > 90, absence of extra-cerebral metastases, and no history of ALK inhibitor treatment before the development of BM were associated with improved OS. In a small retrospective review, Choi et al. demonstrated that SRS combined with crizotinib yielded excellent LC, especially for patients with oligometastatic disease (12 months PFS—84.2%). Only one patient required hospitalization due to brain edema after SRS and was treated with corticosteroids alone. However, to date, no level 1 evidence supporting this combinatorial approach has been generated.

#### 3.2.4. Angiogenesis Inhibitors

Antiangiogenic drugs, such as bevacizumab, block vascular endothelial growth factor (VEGF), which inhibits tumor angiogenesis and reduces intratumoral hypoxia, making them potential RT sensitizers. Low doses of RT facilitate tumor growth and metastasis in mouse models by increasing angiogenesis and activating VEGFR2 [53]. Wang et al. prospectively evaluated the combination of SRS and bevacizumab for the treatment of BM with extensive edema. Bevacizumab was administered between 3–10 days after completion of SRS for a minimum of two cycles (5 mg/kg, at 2-week intervals) [54]. They reported no severe toxicity, and no recurrent edema or RN. Yomo et al. reported on salvage SRS with adjuvant bevacizumab for heavily pre-treated recurrent BM [55]. The first cycle of bevacizumab (7.5–10 mg/kg intravenous) was given after salvage SRS. With a median of 4 (range 2–10) cycles of bevacizumab, no neurotoxicity was reported, and the combination provided adequate radiographic response and neurologic palliation. Guinde et al. reported no systemic or cerebral adverse events in patients with NSCLC BM receiving SRS, and bevacizumab [56]. Once again, the quality of these data and the level of evidence remains weak, and well-designed clinical trials are desperately needed in this domain.

#### 3.2.5. BRAF Inhibitors

The mitogen-activated protein kinase (MAPK) pathway is often upregulated in cancer cells, and it is also activated by exposure to ionizing radiation [57,58]. Multiple retrospective studies have demonstrated high efficacy rates of BRAF inhibitors combined with SRS for patients with melanoma BM, overcoming the preconceived notions of radioresistance [59,60]. In a prospective study involving 80 patients with melanoma BM, SRS combined with BRAF inhibitors was found to increase OS (median OS 11.2 vs. 4.5 months) compared to SRS alone [61]. A multicenter retrospective cohort study reported on patients with melanoma BM treated with SRS and BRAF inhibitors [62]. The asymptomatic intracranial bleeding rate in the SRS plus BRAF inhibitor group was 10.4% vs. 3% in the group without the BRAF inhibitor (*p* = 0.03). On the other hand, other studies have reported the bleeding rate to be very low, approximately 2.8%, in patients treated with SRS and BRAF inhibitors [63,64]. In summary, low-level evidence is available to suggest that the combination of SRS and BRAF inhibition improves LC and OS, but with the possibility of increased intratumoral bleeding. Randomized clinical trials are required to define the risks and benefits more clearly.

#### 3.2.6. MEK Inhibitors

The MEK inhibitor trametinib is usually combined with BRAF inhibitors. Patel et al. reported the initial experience of the combination of BRAF and MEK inhibition with SRS for BRAF-mutant melanoma BM with six patients being treated with SRS within 3 months of BRAF and MEK inhibitor administration [65]. The median OS was 23.1 months from the date of BRAF and MEK inhibitor administration. There was no evidence of increased or unexpected toxicity with the addition of SRS. In another study, 39 patients received a BRAF inhibitor ± trametinib concurrently with SRS, and the median PFS was 12.7 months (95% CI: 8.3–18.5) [66]. Since the number of patients treated with MEK and SRS was small in both the studies, definitive recommendations about safety cannot be drawn, and prospective trials are needed.

#### 3.2.7. Cyclin-Dependent Kinase Inhibitors (CDK4/6)

Cyclin-dependent kinase (CDK) inhibitors, namely palbociclib, ribociclib and abemaciclib were recently granted FDA approval and are currently prescribed in combination with hormone therapy to treat hormone receptor positive, HER2 negative metastatic breast cancer [67]. Preclinical data suggest possible synergistic effects with RT [68]. However, data regarding toxicity when combining CDK4/6 inhibitors with RT are scarce. Figura et al. reported retrospective data of breast BM treated by CDK4/6 inhibitors (either palbociclib or abemaciclib) with (43%), before (21%), or after (36%) SRS [69]. There was no increase in neurotoxicity related to the combination therapy. Two lesions (5%) developed RN, both of which had received prior RT. A combination treatment of SRS and a CDK inhibitor appears to be feasible based on this study, but once again the quality and quantity of data is sparse.

Several clinical trials are currently ongoing to evaluate and study the combination of SRS with various targeted agents for patients with BM, as summarized in Table 1.

## 4. SRS and Immunotherapy

**Figure 2:**
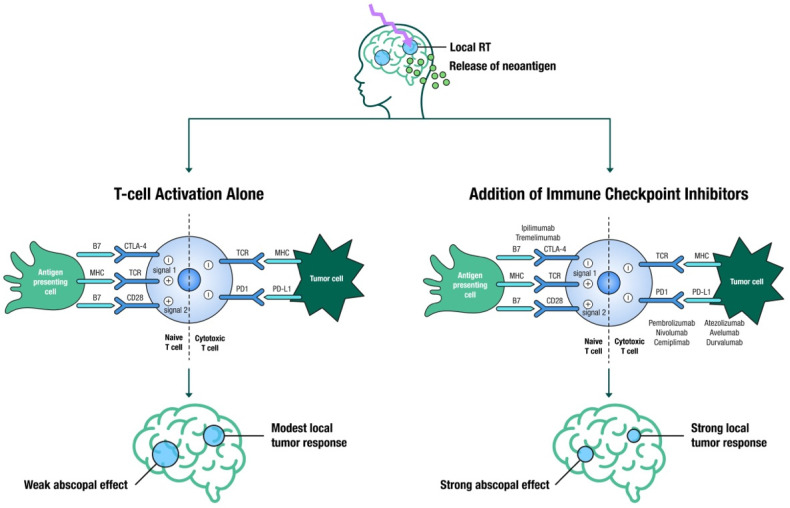
Illustration of the immune stimulatory effects of SRS leading to a localized breakdown and permeability of the BBB, causing the release of tumor associated neoantigens, ultimately leading to T-cell activation by antigen presenting cells (modest local tumor response and a weak abscopal effect); in contrast, the addition of immune checkpoint inhibitors (anti-CTLA4, anti-PD1 and anti-PDL1) to SRS leads to strong local tumor response and a strong abscopal effect (abscopal effect is defined as tumor shrinkage or elimination in sections of the body not directly targeted by local therapy).

SRS is known to increase both innate and adaptive immune responses, making tumor cells more susceptible to T-cell-mediated killing [70] (Figure 2). The aim is to evoke an immune response that will not only boost local effects but also lead to an abscopal response, which occurs outside of the irradiated area [70]. Large registry studies have demonstrated improved OS with SRS and ICIs in patients with BM [71], yet several questions regarding appropriate timing, fractionation, toxicities, and out-of-field responses remain unanswered, and thus several trials are attempting to address these knowledge gaps [72].

### 4.1. Timing and Sequencing

The optimal sequence for these modalities is still unclear, with conflicting published results [72]. Several studies suggest that SRS acts as an antigenic primer by releasing neoantigens from dying cancer cells, and the resultant activated T-cells are further stimulated by ICIs to sustain the immune response. Furthermore, SRS eradicates the inhibitory T-cells in the tumor microenvironment, which would otherwise dampen the immune response [73,74]. This hypothesis would suggest that close temporal sequencing of SRS and ICIs is required. Underscoring this hypothesis, ipilimumab before SRS resulted in a higher partial response rate as compared to ipilimumab administered after SRS (40% vs. 16.7%) [75]. However, a large retrospective study showed that neoadjuvant ICI had no additional advantage over adjuvant ICI [76].

Some studies advocate for the administration of SRS immediately before ICI, with the rationale that activated T-cells in the tumor microenvironment would be killed by SRS. Conceptually, using SRS prior to ICIs would put fewer activated T-cells at risk. In a retrospective analysis of melanoma BM, RT followed by ICI was compared to ICI followed by RT [77]. The RT followed by ICI group had superior survival as compared to the ICI followed by RT group. Another series reported a significantly longer local recurrence-free duration in melanoma BM patients treated with SRS either before or with ipilimumab as compared to patients treated with SRS after completing ipilimumab (median 19.6 months vs. median 3 months) [78].

The concept of concurrent treatment of ICI with to SRS is still up for debate, with some studies using a 2-week window while others extending this to 1 month [79]. Although the timing of SRS in relation to ICIs is likely to be influenced by the agent of choice and its half-life, as well as the mechanism of immune activation and response, it appears that ICIs given four weeks before or after SRS have shown the best results [80]. Prospective studies in BM patients are urgently needed to assess the timing and sequencing of ICIs with SRS (Table 2).

### 4.2. Impact of Corticosteroids

The immunosuppressive effect of corticosteroids may reduce the efficacy of a PD-L1 blockade. Kotecha et al. reported that in patients who received SRS and concurrent ICI, median survival was markedly better with steroid avoidance during the treatment (cumulative dose during and after SRS: 0 mg dexamethasone: 25.1 months vs. ≤60 mg: 10.2 months, *p* = 0.002) [76]. In another multi-institutional study, patients who were PD-L1-naïve with advanced NSCLC were treated with a single-agent PD-L1 blockade [81], and baseline corticosteroid use of ≥10 mg of prednisone equivalent was associated with poorer outcomes. It is recommended that corticosteroids be used with caution before starting ICIs.

### 4.3. Pseudo-Progression and Radiation Necrosis

The synergistic combination of SRS and ICIs also raises concerns about possible side effects, including pseudo-progression and RN [82]. Hubbeling et al. studied adverse radiation effects (AREs)—the imaging correlate of RN in relation to ICI treatment status, RT type, and timing of treatment [83]. They concluded that ICIs and RT did not increase the risk of AREs. On the other hand, Martin et al. evaluated the risk of RN in melanoma, NSCLC, or renal cell carcinoma BM in patients who received a combination of ICIs and RT [84], and discovered a correlation between the occurrence of symptomatic RN and the use of combination therapy, particularly in melanoma patients. Despite reports of an increased risk of RN in some studies, a meta-analysis of the published literature found no evidence of a higher risk than would be predicted with SRS alone [85]. Clearly, the databases for this approach are limited, and of modest quality, given their retrospective nature, and prospective randomized trials are required.

## 5. Future Directions and Conclusions

Recently, the clinical management and understanding of BM has evolved significantly. We have seen a paradigm shift in the management of these patients, and the increased complexity of multi-disciplinary care of these patients only becomes more complicated as clinical practice outpaces evidence development. There remain several key areas of study which are critically needed in clinical trial designs for future studies. First, there needs to be a standardized inclusion of patients with BM on clinical trials testing novel agents, with a key effort to include those with treated or stable disease, active BM, and leptomeningeal disease. This is in line with current recommendations from key society guidelines yet has not been widely adopted. Second, as patients with BM are subgrouped into molecular classes, there is a clear need to understand whether the brain metastasis exhibits the same molecular profile as the primary and, if discordant responses are observed, to develop minimally-invasive means of profiling intracranial disease. Third, there is a critical need to better understand if toxicities in patients treated with combination approaches are related to the systemic therapy, RT, or both treatments. This is unclear in the published literature, given the differences in reporting adverse events or dose-limiting toxicities (typically related to drugs rather than dose-limiting radiation). Fourth, there needs to be a collective effort to homogenize clinical trial designs to better evaluate combination or sequential strategies for BM management. For example, as displayed in Table 2, there are a wide variety of endpoints for the currently available clinical trials, including toxicity, local control, response rate, and survival. Moreover, nine of the ten studies represented are single institution trials, and none of the trials represent phase III randomized studies. Increased cross-institutional effort with multi-institutional and cooperative group designs may allow for larger samples sizes, better external validity of the results, faster accrual, and improved designs to meaningfully impact clinical practice. Finally, as novel immunotherapies, such as anti-CD47 agents, cancer vaccines, and CAR T-cell therapies, are introduced into clinical practice, we recommend careful prospective evaluation of their safety in combination with RT.

## Figures and Tables

**Table 1:** Ongoing trials of SRS and targeted therapies in patients with brain metastasis.

Trial Registration No.	Study Location	Tumor Type	Study Design	Systemic Therapy Agent	*n*	Primary Endpoint	Study Start Date	Estimated Completion Date
NCT04147728	Peking University Third Hospital	NSCLC	Phase II	Anlotinib	50	EI	Dec 2019	Dec 2022
NCT04643847	First People’s Hospital of Hangzhou	NSCLC	Phase II	Almonertinib	47	DOR	Nov 2020	Nov 2023
NCT02726568	Betta Pharmaceuticals Co., Ltd.	NSCLC	Phase II	Icotinib	30	PFS	Mar 2016	Dec 2022
NCT03535363	Case Comprehensive Cancer Center	NSCLC	Phase I	Osimertinib	6	MTD	Oct 2018	Aug 2021
NCT03769103	British Columbia Cancer Agency	NSCLC	Phase II	Osimertinib	76	PFS	Mar 2019	April 2025
NCT03497767	Trans-Tasman Radiation Oncology Group	NSCLC	Phase II	Osimertinib	80	PFS	Aug 2019	March 2024
NCT04856475	Jules Bordet Institute	Breast	Phase II	Neratinib	104	ORR	July 2021	July 2025
NCT03190967	National Cancer Institute (NCI)	Breast	Phase I/II	T-DM1 and Metronomic Temozolomide	125	MTD	April 2018	June 2023
NCT04585724	Emory University	Breast	Phase I	Abemaciclib, Ribociclib, or Palbociclib	25	AE	June 2020	Oct 2021
NCT04074096	UNICANCER	Melanoma	Phase II	Binimetinib and Encorafenib	150	PFS	Sep 2021	Sep 2028
NCT03898908	Grupo Español Multidisciplinar de Melanoma	Melanoma	Phase II	Binimetinib and Encorafenib	38	ORR	July 2019	Oct 2023
NCT03430947	Technische Universität Dresden	Melanoma	Phase II	Vemurafenib and Cobimetinib	20	ORR	July 2018	July 2022
NCT02974803	Canadian Cancer Trials Group	Melanoma	Phase II	Dabrafenib and Trametinib	6	ORR	Nov 2016	June 2021

Abbreviations: *n* = number; NSCLC = non-small cell lung cancer; EI = edema index; DOR = duration of response; PFS = progression-free survival; AE = adverse events; MTD = maximum tolerated dose; RR = response rate; ORR = objective response rate.

**Table 2:** Ongoing trials of SRS and immune checkpoint inhibitors in patients with brain metastasis.

Trial Registration No.	Study Location	Tumor Type	Study Design	Immunotherapy Agent	*n*	Primary Endpoint	Study Start Date	Estimated Completion Date
NCT03483012	Dana-Farber Cancer Institute	Breast	Phase II	Atezolizumab	45	PFS	Sep 2021	Sep 2025
NCT03449238	Weill Medical College of Cornell University	Breast	Phase II	Pembrolizumab	41	RR, OS	Nov 2018	Dec 2026
NCT03807765	H. Lee Moffitt Cancer Center and Research Institute	Breast	Phase I	Nivolumab	14	DLT	Jan 2019	Jan 2022
NCT02886585	Massachusetts General Hospital	Any solid tumor	Phase II	Pembrolizumab	102	RR, OS	Oct 2016	Sep 2022
NCT02097732	University of Michigan Rogel Cancer Center	Melanoma	Phase II	Ipilimumab	40	LC	April 2014	July 2020
NCT03340129	Melanoma Institute Australia	Melanoma	Phase II	Nivolumab & Ipilimumab	218	NSCD	Aug 2019	Aug 2025
NCT03297463	Masonic Cancer Center, University of Minnesota	Melanoma	Phase I/II	Ipilimumab	40	MTD, ORR	Jan 2018	Feb 2020
NCT02716948	Sidney Kimmel Comprehensive Cancer Center	Melanoma	Phase I	Nivolumab	90	AE	Jun 2016	Mar 2023
NCT02858869	Emory University	Melanoma, NSCLC	Phase I	Pembrolizumab	30	DLT	Oct 2016	Oct 2021
NCT02696993	M.D. Anderson Cancer Center	NSCLC	Phase I/II	Nivolumab & Ipilimumab	88	DLT, PFS	Dec 2016	Dec 2020
NCT02978404	Centre hospitalier de l’Université de Montréal (CHUM)	NSCLC, RCC	Phase II	Nivolumab	26	PFS	Jun 2017	Jun 2022

*n* = number; NSCLC = non-small cell lung cancer; RCC = renal cell carcinoma; OS = overall survival; PFS = progression-free survival; DLT = dose limiting toxicity; AE = adverse events; LC = local control; MTD = maximum tolerated dose; RR = response rate; ORR = objective response rate; NSCD = neurological specific cause of death.
